# “Gateway hypothesis” and early drug use: Additional findings from tracking a population-based sample of adolescents to adulthood

**DOI:** 10.1016/j.pmedr.2016.05.003

**Published:** 2016-05-28

**Authors:** Stephen Nkansah-Amankra, Mark Minelli

**Affiliations:** a Sam Houston State University, College of Health Sciences, Department of Population Health, 241I CBS Building, Huntsville, TX 77340, United States; b Central Michigan University School of Health Sciences, Health Professions Building, Room 2235, Mt. Pleasant, MI 48859, United States

**Keywords:** Gateway theory, Gateway drugs, Depressive symptoms, Early drug use, Young people

## Abstract

To evaluate the consistency of the relationship between early drug use in adolescence and illegal drug use in adulthood as proposed in the “gateway theory” and to determine whether pre-existing depressive symptoms modifies this relationship. We used contractual data from the National Longitudinal Study of Adolescent to Adult health data spanning a 14 year period. We assessed the relationship between gateway drugs at baseline (age 11–20 years) and drug use in adulthood using generalized estimating equation (GEE) regression models. Gateways drugs used in early adolescence were significantly associated with marijuana use, illegal drugs and cocaine in older adolescence, but over time these relationships were not consistent in adulthood. Changes in the pattern of psychoactive drug use were important predictors of drug use in adulthood. A history of higher depressive symptoms was associated with higher frequencies of psychoactive drug use over time. Users of mental health services in adolescence were less likely to use drugs in older adolescence and in adulthood. Relationships between early drug use and later drug use in adulthood cannot be solely explained by the gateway hypothesis. Collectively, adolescent drug prevention and treatment programs should apply theory-based and evidence-proven multisectoral intervention strategies rather than providing a brief counseling on individual's behaviors. This evidence should include understanding that changes in behavior should involve broader analyses of the underlying social context for drug use and in particular the role of the community social norms in driving a group's behaviors.

## Highlights

• Evidence shows early use of legal drugs increase risks of illicit drug progression.
• It is unclear if adolescents at different stages, experience drug use in adulthood.
• We used a nationally-based longitudinal data to evaluate these relationships.
• Early drug initiation increased risks of drug progression but not in adulthood.
• GH might be inadequate to explain drug use over time.

## 1 Introduction

The concept of “gateway hypothesis” has been studied since the 1970s (Kandel, 1975, Kandel and Faust, 1975) as the theory suggests that an adolescent's early experimentation with alcohol or tobacco or cannabis escalates to more addictive illicit drugs later in adulthood (Lynskey et al., 2003). Most commonly used illicit substances include heroin/opioids, cocaine and or amphetamines and their designer drug analogs, considered illegal by the criminal justice system in the United States and other jurisdictions. Early onset or drug experimentation has been elaborated and characterized in distinct pathways in the substance abuse and dependence literature. Overall, the theory has had mixed results showing both a link or sequence of licit drug use to illicit drug use (Guxens et al., 2007, Guxens et al., 2007, Korhoene et al., 2010, Lessem et al., 2006, Mayet et al., 2012) and no association (Mackesy-Amiti et al., 1997, Golub and Johnson, 1994).

Although the concept has also been a subject of considerable scholarly and political discourse in western societies, a review of the literature often shows less consensus on research and policy relevance among investigators. An earlier series of studies (Kandel, 1975, Kandel and Faust, 1975, Kandel et al., 1992) among adolescents showed the existence of a significant and a clearly defined sequence of drug use onset starting with licit substances (alcohol, cigarette) and progression to illicit drugs (cocaine, marijuana, methamphetamine, and heroin) through adulthood. Recently, Kandel and Kandel (2014), have demonstrated the GH with animal studies and their findings showed that use of one drug enhances effects of the other drugs — a process hypothesized as due to the priming of the neural circuitry of the brain. Fergusson et al. (2006) analyzed a population-based data on cannabis use and progression to other illicit drugs among a 25-year longitudinal study of 1265 birth cohorts from Christchurch, New Zealand. The investigators found strong evidence for causal model of GH, in which earlier use of cannabis was hypothesized as causing increased use of other illicit drugs. In addition, numerous prior studies have failed to disconfirm causal links of gateway effects in human populations (Kandel, 2002, Gundy and Rebellon, 2010, Morral et al., 2002). However, a cross-country comparison of the GH by Degenhardt et al. (2010) found background prevalence of the gateway drugs or their availability as the major driving factor for drug use progression across countries analyzed. Another study analyzing a sample of adolescents from South Florida showed that marijuana gateway effect is contingent on context of age (Gundy and Rebellon, 2010). In spite of these, it is still unclear the extent to which a cohort of adolescents at different developmental stages experience gateway drug use (tobacco, alcohol, marijuana) as determinants of later illicit drug use during and prior to adulthood.

Consistent with the theory, research in the substance abuse literature has focused on age of onset of substance used as a proximate determinant of future drug use and dependence in adulthood (Chen et al., 2009, Behrendt et al., 2009, Trenz et al., 2012, Mayet et al., 2012). Trenz et al. (2012) found early onset of alcohol at age 15 but not cigarette or marijuana among adolescents as a risk factor for injection drug use in adulthood. An earlier study by Chen et al. (2009), reported clinical manifestation of drug dependence and other health problems among adolescents' early onset (11–17 years) of drug use compared with adult (18 +) recent users. Likewise, Lynskey et al., report that among discordant twins, individuals starting cannabis use before age 17 were at increased risk of illicit drug use and drug dependence. Mayet et al. (2012) found that among 17-year olds participating in a military exercise in France, initiating one drug increased the risk of initiating the other drug use, consistent with the gateway theory. However, the risk of an experimenter becoming a daily user of tobacco was higher for initial tobacco users than cannabis. A recent follow-up study by Mayet et al. (2012) found daily tobacco use among adolescents as strongly associated with cannabis initiation and other illicit drugs. However, deviations to these patterns of drug use have been also observed in studies and hypothesized to be linked to an underlying mental health condition of respondents (Degenhardt et al., 2010). One study of cross national comparisons of 17 countries found prior drug use and age of onset as the most dominant factors determining drug dependence (Degenhardt et al., 2010). In addition, there were considerable variations of early onset of drug use among similar age cohorts in different countries. Unfortunately the prospective relationship between early drug onset in adolescence and drug use transition in adulthood was not evaluated.

Although the evidence suggests that substance use dependence may also occur with the initial drug experimentation of commonly available legal substances (Kirby and Barry, 2012), continual use over time may increase the likelihood of developing risks for substance use disorders (Deza, 2015) and other substance-related illnesses. In this regard, Midanik et al. (2007), reported that simultaneous alcohol and cannabis use was related to increased prevalence rates of other social consequences including problem behaviors, alcohol dependence and depression. A twin-study of young women by Agrawal et al. (2009), found that women's initial use of tobacco and cannabis simultaneously was more likely to experience higher rates of DSM IV cannabis abuse but not dependence. The possibility of other addictive drugs (codeine and other prescription drugs) and substances (hallucinogens, inhalants, ecstasy, amphetamines) resulting in poor health sequelae due to initial drug experimentation has been noted in some studies (Fairman, 2015, Deza, 2015). This is important considering the extent of initial substance use or use combinations could lead to more widespread illegal drugs or addictive behaviors over time, replications of these findings in a nationally representative sample of adolescents transitioning to adulthood are needed to understand the continuum of progression of drug use over the life course (from adolescence to adulthood).

Despite a great uncertainty about the gateway theory, with few exceptions (Behrendt et al., 2009, Fergusson et al., 2006) there has been remarkably less rigorous empirical assessment with a population-based sample, prospectively assessing the impact of early drug use on later drug use as well as related mental health conditions (depressive symptoms). Data from longitudinal studies will allow for additional questions to be explored including how changes in drug use over time from early adolescence to adulthood might be related to earlier onset of drug use and a pattern of individual drug use trajectories during transition to adulthood. Sequence of drug initiation may be due to several factors including effects of one drug use on another, familial and demographic and psychosocial characteristics or a combination of different factors (Guerra et al., 2000). In addition most of these studies did not control for current substance use, a factor which is an important determinant for fully understanding how earlier drug use or non-drug use may change over time from adolescence to young adulthood.

The aim of this study is to evaluate the impact of early substance use on later illicit drug use while accounting for concurrent drug use over a relatively longer period among a cohort of adolescents transitioning to adulthood, and to determine the extent to which these relationships conform to the GH. Our analyses here examine the relationship between early gateway drug use and future illicit drug use among a cohort of adolescents, and to determine whether causal or non-causal inferences are warranted. We anticipate gateway drug use among our sample to escalate to illicit drug use in adulthood and we expect this relationship to be non-causal. Our hypothesis is that any gateway relationship in adulthood reflects spurious effects of underlying depressive symptoms and age as well as modifying influences of these factors (age and depressive symptoms). Second, we were also interested in investigating the relationship between early drug use onset in adolescence and substance use in adulthood taking into account the existing concurrent mental health status of individuals at each developmental stage. To the extent that gateway associations to illicit drug use among older adolescence or adulthood is causal, we evaluate the stage (older adolescence or adulthood) at which this relationship is likely to significant and if it has short or long term effect in adulthood. We hypothesize that the gateway relationship to adult drug use is transient only among older adolescence and the relationship is modified by depressive symptoms reported in older adolescence.

## 2 Materials and methods

The study sample was generated from the National Longitudinal Study of Adolescent to Adult Health (Add Health). The Add Health study is a national longitudinal survey of school-based representative sample of students in grades 7–12 in 1994 academic year in the United States. The cohort from this study were selected and interviewed in 1994–95 school year as in-home samples (79% response rate as a proportion of selected in-home sample) for Wave I (*N* = 20,745). This sample has been followed over time with three further in-home interviews in 1996 Wave II (11–21 years, response rate 88.6%; *N* = 14,738), Wave III 2001–2002 (aged 18–26 years, response rate 77.4%; *N* = 15,197) with the most recent data occurring in Wave IV 2007–08 (aged 24–32 years, response rate 80.3%; *N* = 15,701). Our analysis used the restricted Add Health datasets, and the detailed description of the study design is found in other publications (Bearman et al., 1997, Nkansah-Amankra et al., 2012). In this analysis, we a used a total sample of 11,194 observations with complete survey weights and information across all four waves. The study was approved by the Institutional Review Board (IRB) of Central Michigan University.

### 2.1 Outcome measures

We used the following illegal substances from Waves II to IV as our outcome measures: marijuana, illicit drugs (Add Health instrument gathered information specifically on heroin, amphetamines, LSD, PCP, ecstasy, speed, ice to assess the illicit drugs variable) and cocaine. At each wave of data collection, participants were asked if they had used each of the above substances in the past 30 days. We created a two-level outcome measure for each psychoactive substance used from older adolescence (Wave 2) to adulthood (Waves 3 and 4). Respondents reporting not using a substance served as the reference.

### 2.2 Exposure

The exposure variables of interest were the three known “gateway substances” used in early adolescence (as measured in Wave I): tobacco, alcohol and marijuana. These were measured with two items, one stating if respondents had used any of these substances and the follow-up question asking the age at which they started using each substance for the first time. These two items were combined to create the age of use for the following substances: tobacco, marijuana, and alcohol as ≤ 10, 11–15, and 16–18 consistent with previous investigations (Tarter et al., 2012). We were also interested in examining age of onset of illicit drugs and Cocaine use in this age groups (≤ 10, 11–15, and 16–18). In a separate analysis we specifically examined changes in the gateway drug exposures in adolescence and changes in the pattern of illegal drug use in adulthood.

### 2.3 Covariates

These include age ranges 12–19, 18–26 and 26–32 (at respective Waves 2, 3 and 4), race (Black, White and Hispanics) and current substance used (marijuana, illegal drugs and cocaine) at a particular survey period to control for potential influences on early drug use and later substance use. We used the Center for Epidemiologic Studies of Depression (CES-D) scale as the measure of depressive symptoms and an indicator of mental health condition (Radloff, 1977), but Waves III and IV used only 9-items. Each response from the original item scale was coded as 0–3 (0 = rarely or none of the time, 3 = most of the time), and four-positively formulated items in the original scale were reverse coded to enhance comparability in calculating the summative score. Higher CES-D scores indicate negative emotions or negative affect. The consistency of the 9-item scale in measuring depressive symptomatology has been affirmed in numerous studies (Levine, 2013, Zhang et al., 2012). Across all four waves we created comparable 9-item CES-D scales to assess depressive symptoms. Response to mental using the mental health services item in the instrument was used as a measure of access mental health services across waves of data collection.

## 3 Statistical analysis

All statistical analyses were estimated using SAS Callable SUDAAN version 9.0 (SAS Institute, Cary, NC and RTI, Cary, NC) to account for sampling weights and other survey characteristics in determining the standard errors. Statistical significance for unadjusted comparisons was assessed by using Rao Scott χ^2^ tests. We evaluated cohort-specific analysis to determine the relationship between initial drug intake and later illicit drug progression across different waves of data collection. We estimated the odds ratios (ORs) and 95% confidence intervals (CIs) using the baseline drug use and later drug progression using the category of non-users as the reference groups. These models were estimated with generalized estimating equation (GEE) for repeated measures using cumulative logit link function and simultaneously adjusting for multiple covariates. To model the relationship between early substance used and later substance use as exhibiting a change over time, the variation in early exposures and changes in later drug use escalation were examined. Analyses were 2-sided and p-values < .05 were considered statistically significant.

## 4 Results

Table 1 shows the distribution of baseline mean age, age at first substance use and other socio-demographic characteristics of Add Health participants, 1994/95. The age group ≤ 15 years consistently reported higher percent frequency distribution of substance use in Wave I.

**Fig. 1:**
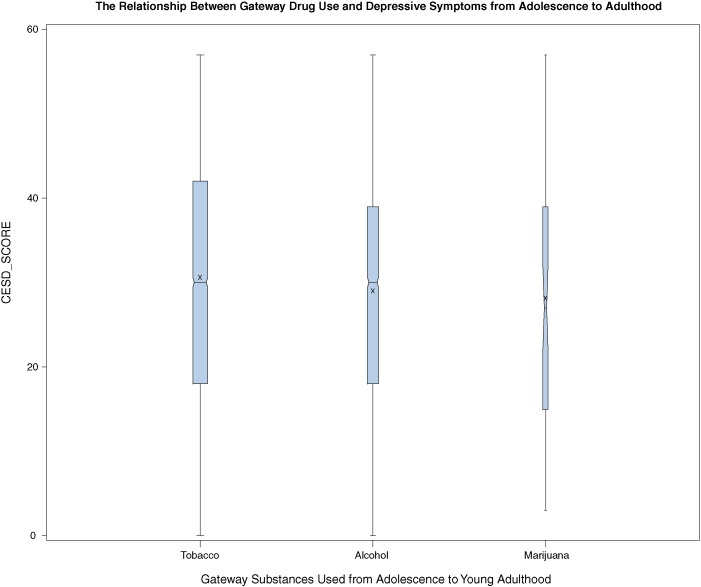
The Relationship between Gateway Drugs Used and Depressive Symptoms from Adolescence to Adulthood.

Fig. 1 shows a box and whisker plot of depressive symptomatology (median, 25th–75th percentiles) participants reporting using separately each gateway drug from Waves I to IV. These plots reveal strong correlations among cigarette smoking (60.8%) or alcohol use (38.5%) and reporting of higher depressive symptoms over time. That is, gateway substance users are over time more likely to report depressive symptoms (as measured with CES-D). However, both cigarette smokers and alcohol users are over time more likely to report relatively higher depressive symptoms than marijuana users.

Associations of baseline characteristics with psychoactive substance use over the period of follow-up data are shown in Table 2. Age group 11–15 years or below reported the highest frequency of drug use over time, compared to other age groups. More than three-quarters of the sample using alcohol in Wave I (11–15 years) reported using all types of illicit drugs over time, but a little more than half of tobacco in Wave I used different illicit drugs. More than half of marijuana users in this age category used marijuana a year later but the usage of other illicit drugs was not consistent over time. Overall, illegal drugs and cocaine in particular were least likely to be used from adolescence to adulthood.

Relationships among various psychoactive substance uses with the baseline age of substance use measured at 3 different survey waves are shown in Table 3. The first three columns of Table 3 show odds ratio (OR) estimates and corresponding 95% confidence intervals (95% CI) for predicting drug use and mental health services access among older adolescents. Tobacco, marijuana, any illegal drugs and age at cocaine use in adolescence was significantly associated with marijuana use, illegal drugs and cocaine in older adolescence, but over time these relationships were not consistent as expected from the gateway hypothesis. Using marijuana at baseline appeared to be consistently associated with increased likelihood of using other psychoactive substances in late adolescence and in young adulthood compared with non-users. Alcohol use in Wave I was less likely to be associated with any psychoactive substances in older adolescence and over time, but tobacco use greatly increased the odds of using marijuana, cocaine and illegal drugs in older adolescence. Cigarette smoking greatly increased the odds of using cocaine in early adulthood among all age groups reporting smoking in Wave I.

The pattern consistent with the gateway hypothesis was not present across the waves of data collection in to adulthood. However, among the three gateway substances initiated in early adolescence marijuana appeared somehow to have a greater and consistent effect in determining the likelihood of using other psychoactive substances over time in adulthood.

There were significant interactions between the three gateway drugs and depressive symptoms for marijuana, illegal drugs and cocaine used in older adolescence and adulthoods (results not shown). Age groups 11–15 years smoking cigarette in Wave 1 and reporting high depressive symptoms (in Wave I) increased the odds of smoking marijuana in older adolescence (OR = 1.37; 95% CI = 1.08, 1.74) and young adulthood (OR = 1.54; 1.10, 2.16), whereas age groups 16–18 smoking cigarette in Wave I and reporting high depressive symptoms in Wave 1 were at higher odds for illegal drug use in older adolescence (OR = 10.08; 95% CI = 1.59, 63.96).

Table 4 shows results from changes in the use of three gateway drugs in adolescence and the likelihood of using illegal substances in adulthood. Controlling for all potential confounders (race, age and current drug use) persistent smoking in adolescence was associated with increased odds of marijuana use in early adulthood, and marijuana (OR = 1.47; 95% CI = 1.17, 1.83), illegal drugs (OR = 3.28; 95% CI = 2.73, 3.94) and cocaine (OR = 3.70; 95% CI = 3.09, 4.44) in young adulthood. Only heavy alcohol users were at increased odds of using marijuana in early adulthood and higher odds of using illegal drugs and cocaine in young adulthood.

We next investigated whether the observed effects resulting from the changes in the gateway drug use in adolescence and depressive symptoms (CES-D) were consistent determinants of illegal drug use in adulthood. Non-smokers in Waves 1 and 2 and reporting high depressive symptoms in Wave 3 had 1.5 times the odds of smoking marijuana in early (OR = 1.52: 95% CI = 1.11, 2.08) and young (OR = 1.55 95% CI = 1.11, 2.16) adulthoods but lower risk of using illegal drugs in early adulthood (OR = 0.29, 95% CI = 0.13, 0.66). Current smoking status in both waves and reporting elevated depressive symptoms in Wave 2 increases the odds of using illegal drugs in early adulthood (OR = 2.22, 95% CI = 1.12, 4.40), or smoking marijuana in young adulthood (2.32 (95% CI = 1.52, 3.56). But those quitting smoking in Wave 2 and reporting high depressive symptoms in Wave 2 had more than 24 times the odds of using illegal drugs in early adulthood (OR = 24.51, 95% CI = 1.87, 322.02). Individuals taking alcohol either in Wave 1 or Wave 2 (fluctuating drinkers) and reporting low depressive symptoms in Wave 1 were at increased odds of smoking marijuana in Wave 3 (OR = 4.41; 95% CI = 1.12, 17.34).

## 5 Discussion

In this prospective cohort study, early use of psychoactive substances — smoking cigarette, alcohol and illegal drugs (as earlier defined) was associated with increased likelihood of using marijuana, illegal drugs and to a large extent cocaine use in older adolescence. First, early exposure to marijuana and illegal substances was also positively associated with illegal substance and cocaine use in young adulthood. Second, cocaine use in early adolescent appeared uniquely to have ‘a long reach’ in later cocaine use in young adulthood. However, over time from adolescence to adulthood, we did not observe a pattern where early exposure to commonly known psychoactive substances — cigarette smoking or alcohol escalates to marijuana use or illegal psychoactive substances as posited by the ‘gateway theory.’ Finally, interactions between the gateway drugs and reporting high depressive symptoms in adolescence or adulthood were associated with increased use of marijuana, illegal drugs and cocaine in early or young adulthood.

Our finding that early exposure to cigarette smoking and alcohol use was positively associated with later (almost 10.4 months) use of illegal psychoactive substances among older adolescence is consistent with numerous studies on the gateway hypothesis (Kandel, 2002, Agrawal et al., 2009, Mayet et al., 2012). However, our findings showed that over a relatively longer period of time (from adolescence to adulthood), early use of marijuana and other illegal drugs rather than tobacco or alcohol greatly increases the likelihood of using cocaine and other illegal drugs. A co-twin study in Australia found early cannabis use as a consistent predictor for other psychoactive substance use and in development of drug dependence (Lynskey et al., 2003).

Contrary to our findings, Tarter et al. (2012) did not find early drug use of gateway drugs (tobacco, alcohol) as predicting marijuana and other illicit drug use. Participants in this study started using marijuana before tobacco or alcohol. However, this prior finding reflects ease of access to marijuana or other commonly available drugs rather than a defined pattern of drug escalation within a framework of causality. This needs further investigations.

Our data reveal that early use of psychoactive substances is associated with increased likelihood of using further illicit substances during adolescent period, but effects of these substances on later illicit drug use are inconsistent. However, early use of marijuana also appears to more readily ‘open the gate’ towards later use of other illicit substances. These findings are remarkable in view of the current debates on legalizing marijuana for recreational and medical uses, and the fact that our sample is population-based. Clearly, marijuana use in early adolescence enhances increased likelihood of continuing use of other psychoactive substances, and may be further compromised by underlying mental health condition. Existing drug policy and intervention programs have placed more emphases on tobacco, alcohol as ‘gateway’ drugs to later illicit drugs, but our findings suggest that attention should equally be placed on marijuana and other psychoactive substances in some population groups particularly in the age groups ≤ 15.

Our findings also reveal that it is not solely early exposure to psychoactive substances that matters for later drug use, but also the *timing* of the exposure to these ‘gateway drugs.’ For some illegal drug use outcomes, particularly those related to marijuana use, alcohol and to some extent tobacco exposures in adolescence may be especially harmful in young adulthood. Both heavy and moderate users of alcohol as well as adolescents using marijuana (of different amounts) in Waves I and II were at increased odds of using illegal drugs and cocaine in young adulthood. The construct of “gateway theory” or GH has some heuristic and intuitive appeal to the academics, policy makers and the general public. The idea of gateway substance use among adolescents actually assumes that once consumption of psychoactive substance is initiated the trend is to escalate and suggests that adolescent behaviors are immutable. Even though this is appealing, the idea is inconsistent with age-related reductions in drug use observed in the human development phenomenon described as ‘maturing out’ (Lee et al., 2013) during emergent adulthood (Littlefield et al., 2009).

Strengths of this study include the length (≥ 14 years) of the Add Health data and the sampling procedure used allowed prospective analyses of variations in different psychoactive drugs used from early adolescence to young adulthood. Availability and inclusion of current drugs used in statistical models across each wave enabled ascertainment of effects of earlier drugs used on current illicit drugs (while controlling for previous drugs). Given the relatively large sample size, we were able to model changes in drug use during adolescence and likelihood of using other drugs in adulthood — a feature that has not been applied in numerous studies. Another unique contribution of this study was analyses of interactive effects among early drug use, depressive symptoms (mental illness) on the risk of later drug use in adulthood, features which were not available in other studies. However, our study has some limitations. First, the taxonomy used in classifying drugs is purely based on the legal status, and social acceptability, not necessarily on the basis of the inherent harm each is likely to cause. Ideally, classifying drugs on dimensions of harmfulness or increased likelihood of addiction and potential size of a threat to the individual and the larger society should be the focus of future investigations. Second, we could not measure amounts of early psychoactive substances that precipitated the use of ‘hard and illicit’ drugs over time. It is almost impossible to evaluate or predict with certainty the quantity of each psychoactive substance(s) needed to achieve these changes. Third, our study could not also examine other psychological and pathological variables associated with early or continuing drug use. In particular, it is not clear whether individuals using marijuana to reduce anxiety in social circumstances such as to avoid certain negative social affects (Buckner et al., 2011). Fourth, if unobserved environmental or genetic factors are associated with early psychoactive substance use and follow-ups, then our model estimations might be biased, and in that case it might be inappropriate to assume that later drug use is solely attributable to early drug use. Finally, historical and secular trends occurring in the use of licit or illicit substances in the country might suggest that describing substance use in terms of onset might be too simplistic because such an account might not consider substance use history over time.

## 6 Conclusion

In conclusion, this study did not find that the proportion of the population using alcohol, tobacco or marijuana in early adolescence showed patterns of increasing use of marijuana, illegal drugs or cocaine according to the length of follow-up (approximately 14 years). These findings suggest that adolescent drug prevention and treatment programs should apply proven multi-sectoral prevention strategies rather than providing brief counseling methods only on individual behaviors. While individual behavior change is desirable, a focus on the individual may be inconsequential compared to radical changes that may need to be made at the broader societal contexts. In addition, such efforts must not only focus on licit substances but include marijuana and assess the underlying mental illness predisposing young people to early drug use. In particular adolescents' recreational use of marijuana needs to be discouraged at the earliest age and medical marijuana use must have strict adherence to treatment regimen.

## Figures and Tables

**Table 1:** Baseline characteristics of Add Health study participants according to early drug use, 1994–2008.

Characteristics	n^a^	%^b^
Race		
Black	2988	20.5
White	5426	61.2
Hispanic	2768	18.3
Sex		
Male	4074	38.5
Female	7120	61.5
Age (years)		
11–15	7716	73.7
16–19	3455	26.3
Cigarette smoking		
None	4185	75.5
1–9	677	12.7
10–19	275	5.4
≥ 20	258	6.6
Alcohol use		
None	2346	23.9
2 days or once a month	805	8.2
1–5 days in past 12 months	6740	67.8
Marijuana		
None	1809	76.0
1–9 times used in past month	610	24.0
Age of onset (years) of cocaine use		
None	10,546	99.4
11–15	14	0.1
16–18	74	0.6
Age of onset (years) of other illicit drugs^c^		
None	10,094	98.3
11–15	26	0.3
16–18	160	1.4
Used mental health services^d^		
No	9661	85.3
Yes	1516	14.7

a Unweighted sample distribution.

b Weighted percentage distribution.

c This variable refers to the use of LSD, amphetamines or heroin and their derivatives and any other illicit more active psychoactive substance.

d Used of mental health services (No = nonuse of mental health services; Yes = reported using mental health services).

**Table 2:** Distribution of population characteristics by early psychoactive drug use^a^ among Add Health participants, baseline in 1994, follow-up in 1996–2008.

	Drug use at respective wave of data collection								
	Wave 2			Wave 3			Wave 4		
Marijuana	Illegal drugs^b^	Cocaine	Marijuana	Illegal drugs^b^	Cocaine	Marijuana	Illegal drugs^b^	Cocaine
	Mean %††	Mean %††	Mean%††	Mean %††	Mean %††	Mean %††	Mean %††	Mean %††	Mean %††
Demographics									
Mean age, years	16.6^c^ 30.25	16.7^c^ 6.2	16.9^c^ 1.9	22.0^c^ 66.9	21.8^c^ 54.0	22.0^c^ 52.0	28.6^c^ 47.9	28.6^c^ 18.1	28.6^c^ 18.8
Age of baseline drug use									
Tobacco (n = 2887; %††)									
Wave 1 non-users	14.1 (408)	8.3 (55)	12.9 (19)	17.9 (338)	23.9 (72)	12.8 (39)	18.7 (253)	15.3 (195)	14.0 (187)
≤ 10	18.2 (411)	18.9 (93)	10.7 (21)	16.1 (271)	9.5 (67)	22.5 (56)	18.8 (261)	17.2 (234)	17.3 (237)
11–15	59.2 (1792)	66.0 (440)	71.8 (151)	67.6 (1232)	54.0 (264)	60.2 (275)	57.1 (935)	63.5 (972)	64.4 (988)
16–18	9.8 (96)	6.7 (34)	3.9 (5)	7.2 (146)	3.6 (31)	4.5 (26)	5.3 (123)	3.8 (89)	4.3 (92)
Alcohol (n = 2129; %††)									
≤ 10	13.3 (273)	13.2 (69)	15.1 (34)	11.8 (160)	5.1 (34)	10.9 (47)	10.9 (115)	10.8 (115)	11.6 (122)
11–15	78.0 (1628)	80.4 (395)	83.0 (126)	81.6 (1049)	89.7 (241)	81.0 (224)	81.0 (814)	84.5 (858)	82.9 (840)
16–19	8.7 (228)	6.4 (38)	1.9 (3)	6.5 (110)	5.3 (34)	8.1 (25)	11.0 (115)	4.7 (80)	5.5 (82)
Marijuana (n = 3330;%††)									
Wave 1 Non-users	30.5 (834)	23.5 (112)	13.0 (24)	43.8 (818)	42.5 (163)	34.1 (127)	37.7 (608)	38.7 (558)	37.3 (525)
≤ 10	3.4 (108)	3.5 (38)	8.5 (22)	3.2 (78)	3.1 (27)	2.5 (27)	3.2 (52)	3.4 (49)	3.7 (49)
11–15	53.8 (1534)	62.5 (413)	63.5 (130)	44.8 (893)	51.1 (209)	55.3 (197)	47.6 (714)	47.4 (717)	47.8 (757)
16–18	12.3 (396)	10.5 (59)	15.0 (17)	8.2 (196)	3.3 (34)	8.1 (44)	11.4 (195)	10.5 (161)	11.2 (169)
Any illegal drugs (3326; %††)									
Non-users	79.6 (2632)	53.8 (323)	58.8 (106)	83.9 (2079)	86.0 (435)	79.5 (346)	86.4 (1710)	77.2 (1364)	80.8 (1388)
≤ 15	15.8 (523)	32.8 (263)	34.1 (80)	12.7 (313)	11.8 (101)	17.8 (99)	11.0 (219)	17.8 (347)	14.4 (311)
16–18	4.6 (51)	13.4 (98)	7.1 (19)	3.4 (108)	2.2 (16)	2.7 (15)	2.6 (67)	5.0 (105)	4.8 (98)
Age of cocaine use (n = 3330; %††)									
Wave 1 Non-users	91.7 (2985)	80.0 (513)	72.6 (124)	92.0 (2290)	92.4 (487)	89.9 (400)	93.3 (1870)	89.0 (1614)	86.9 (1557)
≤ 15	5.9 (236)	12.3 (125)	17.1 (60)	6.3 (175)	5.9 (50)	7.7 (44)	5.0 (101)	8.2 (148)	9.1 (163)
16–18	2.4 (109)	7.7 (45)	10.3 (23)	1.7 (48)	1.8 (15)	2.4 (17)	1.7 (33)	2.8 (52)	4.0 (72)
Sex (n = 3359; %††)									
Male	38.6 (1260)	34.6 (254)	38.6 (97)	46.7 (1106)	48.9 (261)	53.9 (235)	51.9 (1024)	48.5 (888)	50.8 (870)
Female	61.4 (2099)	65.4 (435)	61.4 (114)	53.3 (1425)	51.1 (295)	46.1 (229)	48.1 (1004)	51.5 (947)	49.2 (939)
Race (n = 3357;%††)									
Black	17.1 (753)	1.9 (24)	2.3 (17)	19.4 (72)	8.7 (120)	5.1 (54)	20.6 (593)	5.6 (153)	4.4 (154)
White	60.9 (1683)	82.4 (487)	68.7 (121)	64.8 (360)	72.2 (54)	77.7 (290)	62.3 (993)	77.7 (1273)	72.1 (1161)
Hispanics	22.0 (921)	15.7 (178)	29.0 (73)	15.8 (124)	19.1 (120)	17.2 (120)	17.1 (439)	16.7 (407)	23.5 (492)
CES-D (2709; %††)									
< 24	41.6 (1126)	42.4 (233)	49.0 (72)	44.9 (1355)	47.3 (300)	43.5 (252)	41.7 (1201)	45.5 (1141)	44.3 (1094)
≥ 24	58.4 (1583)	57.6 (317)	51.0 (75)	55.1 (1311)	52.7 (278)	56.5 (225)	58.3 (1127)	54.5 (996)	55.7 (974)
Access to mental health (3359; %††)									
Wave 1 non-users	80.0 (2722)	74.4 (532)	65.4 (136)	83.0 (2096)	83.1 (445)	83.0 (380)	83.8 (1707)	79.3 (1447)	81.3 (1436)
Users	20.0 (637)	25.6 (157)	34.6 (75)	17.0 (435)	16.9 (111)	17.0 (84)	16.2 (321)	20.7 (388)	18.7 (371)

Add Health = National Longitudinal of Adolescent to Adult Health; CES-D = The Center for Epidemiologic Studies Depression Scale.

n = Sample distribution of main variables and other covariates. Figure in parentheses refers to unweighted distribution and %†† describes weighted per cent distribution.

Significant differences between groups at α = 0.05, tested using χ^2^ tests for categorical variables and analysis of variance for continuous variables.

†† Per cent are weighted to account for sampling weights.

a Categories shown above are not mutually exclusive.

b Illegal drugs include any type of illicit drug such as LSD, PCP, ecstasy, mushrooms, speed, ice, heroin or pills without a doctor's prescription.

c Mean age of respective drug used across different wave of data collection.

**Table 3:** Adjusted odds ratios of all later psychoactive substance use (versus non-use) according to baseline characteristics among National Longitudinal Study of Adolescent to Adult Health (Add Health) participants, baseline in 1994, follow-up in 1996–2008.

	Drug use at respective wave of data collection								
	Older adolescents			Early adulthood			Young adulthood		
	Marijuana	Illegal drug^a^	Cocaine	Marijuana	Illegal drugs^a^	Cocaine	Marijuana	Illegal drugs^a^	Cocaine
	OR (95% CI)	OR (95% CI)	OR (95% CI)	OR (95% CI)	OR (95% CI)	OR (95% CI)	OR (95% CI)	OR (95% CI)	OR (95% CI)
Age of baseline drug use									
Tobacco									
Wave 1 non-users	Reference	Reference	Reference	Reference	Reference	Reference	Reference	Reference	Reference
≤ 10	**1.92 (1.28, 2.88)**	2.03 (0.96, 4.27)	2.00 (0.76, 5.23)	0.86 (0.48, 1.51)	**0.28 (0.09, 0.86)**	2.77 (0.73, 10.50)	0.99 (0.61, 1.62)	1.39 (0.93, 2.09)	1.46 (0.97, 2.21)
11–15	1.33 (0.99, 1.79)	1.72 (0.93, 3.18)	**2.45 (1.13, 5.32)**	1.07 (0.37, 1.50)	**0.17 (0.06, 0.47)**	**3.62 (1.11, 11.79)**	**0.57 (0.38, 0.86)**	0.92 (0.65, 1.30)	1.28 (0.90, 1.81)
16–18	**2.79 (1.76, 4.44)**	**3.16 (1.23, 8.13)**	1.24 (0.36, 4.30)	0.74 (0.37, 1.50)	0.61 (0.11, 3.22)	**11.98 (1.57, 91.54)**	**0.50 (0.25, 0.86)**	**0.45 (0.25, 0.81)**	0.56 (0.31, 1.00)
Alcohol									
≤ 10	Reference	Reference	Reference	Reference	Reference	Reference	Reference	Reference	Reference
11–15	0.70 (0.45, 1.45)	0.70 (0.44, 1.10)	0.65 (0.33, 1.28)	**2.34 (1.43, 3.82)**	1.66 (0.73, 3.78)	2.32 (0.90, 5.96)	1.02 (0.65, 1.60)	1.00 (0.71, 1.45)	0.93 (0.63, 1.36)
16–19	0.89 (0.63, 1.25)	0.54 (0.29, 1.02)	**0.11 (0.02, 0.50)**	1.12 (0.57, 2.17)	0.85 (0.27, 2.70)	1.73 (0.40, 7.41)	0.67 (0.36, 1.24)	**0.35 (0.20, 0.61**)	**0.58 (0.35, 0.96)**
Marijuana Use^†^									
Wave 1 Non-users	Reference	Reference	Reference	Reference	Reference	Reference	Reference	Reference	Reference
≤ 10	**8.36 (5.38, 13.01)**	**7.34 (3.64, 14.82)**	**32.8 (12.00, 89.70)**	1.10 (0.61, 1.98)	0.56 (0.18, 1.72)	0.39 (0.14, 1.03)	1.00 (0.53, 1.87)	**3.64 (1.97, 6.72)**	**2.86 (1.61, 5.06)**
11–15	**9.70 (8.27, 11.38)**	**7.79 (5.91, 10.28)**	**13.37 (6.64, 26.92)**	**1.43 (1.14, 1.79)**	0.74 (0.50, 1.07)	1.10 (0.70, 1.73)	1.06 (0.85, 1.23)	**3.19 (2.65, 3.84)**	**3.68 (3.06, 4.41)**
16–18	**9.27 (7.14, 12.04)**	**5.98 (3.80, 9.40)**	**11.80 (4.38, 31.80)**	1.27 (0.86, 1.88)	**0.31 (0.15, 0.67**)	1.11 (0.49, 2.53)	1.22 (0.85, 1.73)	**3.59 (2.65, 4.85)**	**4.81 (3.58, 6.47)**
Any illegal drugs									
Non-users	Reference	Reference	Reference	Reference	Reference	Reference	Reference	Reference	Reference
≤ 15	**5.53 (4.30, 7.11)**	**9.84 (7.58, 12.78)**	**4.29 (2.42, 7.61)**	1.15 (0.62, 2.14)	**0.34 (0.22, 0.53**)	0.89 (0.48, 1.65)	0.76 (0.57, 1.01)	**4.18 (3.28, 5.32)**	**1.43 (1.06, 1.92)**
16–18	**7.09 (4.00, 12.55)**	**22.30 (14.32, 34.71)**	2.27 (0.98, 5.23)	1.66 (0.57, 4.88)	**0.21 (0.10, 0.44)**	**0.14 (0.04, 0.54**)	0.72 (0.45, 1.16)	**5.68 (3.73, 8.66)**	**2.81 (1.33, 5.93)**
Age of cocaine use									
Wave 1 Non-users	Reference	Reference	Reference	Reference	Reference	Reference	Reference	Reference	Reference
≤ 15	**2.93 (2.11, 4.06)**	**4.00 (2.69, 5.96)**	**9.23 (5.75, 14.83**)	0.75 (0.32, 1.75)	**0.34 (0.15, 0.77**)	**0.46 (0.25, 0.87**)	0.96 (0.66, 1.40)	**2.62 (1.62, 4.23)**	**4.35 (3.21, 5.89)**
16–18	**3.30 (1.71, 6.37)**	**4.43 (2.05, 9.60)**	**19.64 (9.53, 40.45**)	0.61 (0.22, 1.68)	0.81 (0.24, 2.74**)**	**0.30 (0.11, 0.81)**	**0.40 (0.21, 0.79**)	0.74 (0.39, 1.40)	**13.92 (7.97, 24.29)**
CES-D									
Low	Reference	Reference	Reference	Reference	Reference	Reference	Reference	Reference	Reference
> High	1.06 (0.89, 1.26)	0.97 (0.70, 1.34)	0.91 (0.52, 1.59)	**0.77 (0.60, 0.99)**	**0.63 (0.40, 0.99**)	1.32 (0.76, 2.28)	**1.31 (1.03, 1.67**)	0.92 (0.76, 1.12)	0.94 (0.77, 1.14)
Mental health services									
Wave 1 non-users	Reference	Reference	Reference	Reference	Reference	Reference	Reference	Reference	Reference
Users	**1.36 (1.15, 1.63)**	**1.69 (1.24, 2.31)**	1.48 (0.99, 2.21)	**0.78 (0.62, 0.98)**	**0.44 (0.30, 0.65)**	**0.61 (0.39, 0.96)**	**0.75 (0.59, 0.95)**	**1.50 (1.23, 1.82**)	1.20 (0.99, 1.47)

Abbreviations: Add Health is National Longitudinal Study of Adolescent to Adult Health; OR, odds ratio, 95% CI: 95% confidence interval.

Multivariable analyses adjusted for demographic characteristics, access to mental health, and previous wave of psychoactive drug use.

Statistically significant differences between groups at α = 0.05. Bold-faced indicate statistically significant differences.

a Illegal drugs include any type of illicit drug such as LSD, PCP, ecstasy, mushrooms, speed, ice, heroin or pills without a doctor's prescription.

**Table 4:** Adjusted odds ratios¶ and corresponding 95% confidence intervals of changes occurring in using drugs in early or young adulthood among respondents participating in the National Longitudinal Study of Adolescent Health (Add Health), 1994–2008.

	Early adulthood^a^			Young adulthood^b^		
Marijuana	Illegal drug use	Cocaine	Marijuana	Illegal drugs	Cocaine
OR (95% CI)	OR (95% CI)	OR (95% CI)	OR (95% CI)	OR (95% CI)	OR (95% CI)
Exposure changes in Waves 1–2						
Tobacco use						
Non-smokers	Reference	Reference	Reference	Reference	Reference	Reference
Persistent smokers	1.96 (1.56, 2.45)	0.87 (0.57, 1.28)	1.28 (0.79, 2.08)	1.47 (1.17, 1.83)	3.28 (2.73, 3.94)	3.70 (3.09, 4.44)
New onset	3.48 (1.22, 9.91)	6.73 (1.39, 32.61)	1.31 (0.28, 6.08)	0.67 (0.27, 1.69)	1.27 (0.59, 2.70)	1.54 (0.76, 3.11)
Quitters	0.99 (0.71, 1.38)	0.29 (0.11, 0.78)	0.39 (0.16, 0.95)	0.91 (0.64, 1.28)	0.99 (0.72, 1.36)	0.93 (0.67, 1.29)
Alcohol use						
Non-users	Reference	Reference	Reference	Reference	Reference	Reference
Heavy drinkers	1.69 (1.21, 2.37)	0.45 (0.24, 0.86)	1.88 (0.92, 3.85)	0.96 (0.69, 1.33)	1.93 (1.45, 2.57)	1.56 (1.20, 2.04)
Moderate users	1.30 (0.73, 2.32)	0.28 (0.11, 0.70)	0.99 (0.30, 3.24)	0.69 (0.38, 1.22)	1.96 (1.22, 3.13)	1.86 (1.18, 2.94)
Fluctuate/relapse	1.18 (0.92, 1.92)	0.45 (0.16, 1.27)	0.89 (0.24, 3.24)	0.59 (0.35, 0.98)	1.33 (0.85, 2.09)	0.49 (0.30, 0.80)
Marijuana use						
Non-users	Reference	Reference	Reference	Reference	Reference	Reference
1–19	1.05 (0.59, 1.87)	1.31 (0.49, 3.55)	1.41 (0.38, 5.22)	2.50 (1.45, 4.31)	2.14 (1.30, 3.52)	2.50 (1.45, 4.31)
≥ 20	0.73 (0.28, 1.90)	0.92 (0.20, 4.21)	0.35 (0.06, 2.17)	1.20 (0.50, 2.86)	8.07 (4.10, 15.90)	1.20 (0.50, 2.86)
Irregular (1 or ≥ 20)	1.10 (0.74, 1.64)	1.78 (0.82, 3.87)	0.89 (0.36, 2.18)	1.47 (1.02, 2.13)	1.94 (1.37, 2.74)	1.47 (1.02, 2.13)

Abbreviations: CI = confidence interval; OR, odds ratio; Add Health.

CES-D is Center for Epidemiologic Study of Depression Scale.

^§^Initial drug use refers to use of tobacco, alcohol, marijuana (gateway drugs) at baseline of the study, in the early adolescence. Reference group is non-users of a particular gateway drug at the respective developmental stage.

¶ Adjusted for the following covariates at different exposures: age, gender, race,

a Young adulthood (age groups 19-23 years).

b Older adulthood (age groups 24-33 years).
